# Treated but Uncontrolled: Characterizing Hypertension in a Sample of 357 Older Adults in the Southeastern United States

**DOI:** 10.3390/geriatrics10040101

**Published:** 2025-07-26

**Authors:** Rachel Helms, Laura A. Robinson, Paul S. Fiore, Kelly P. Strickland, Sarah O. Watts, Felicia J. Tuggle, Jennifer L. Slay, Jeanna Sewell, Andrew D. Frugé

**Affiliations:** 1 College of Nursing, Auburn University, Auburn, AL 36849, USA; 2 Department of Nutritional Sciences, Auburn University, Auburn, AL 36849, USA; 3 College of Science and Mathematics, Auburn University, Auburn, AL 36849, USA; 4 College of Nursing, East Tennessee State University, Johnson City, TN 37614, USA; 5 Department of Sociology, Anthropology, and Social Work, Auburn University, Auburn, AL 36849, USA; 6 Harrison College of Pharmacy, Auburn University, Auburn, AL 36849, USA; 7 School of Kinesiology, Auburn University, Auburn, AL 36849, USA

**Keywords:** hypertension, older adults, pharmaceuticals, socioeconomic, disparities

## Abstract

**Background/Objectives**: Hypertension (HTN) continues to be a leading cause of death and disability in older adults, especially in the southeastern United States. A cross-sectional study was conducted to evaluate the relationships among measured, diagnosed, and treated (HTN) in community-dwelling adults participating in student-led health screenings in eastern Alabama. **Methods**: Between 2017 and 2019, students from health-related disciplines facilitated screenings at 23 community and independent living sites to conduct health assessments, including measuring blood pressure (BP), obtaining medical history, and evaluating current prescriptions. Statistical analyses including chi-square tests, *t*-tests, and backward stepwise linear regression were performed. **Results**: The current sample includes data from 357 adults aged 60 to 99 years (mean age 74.6 ± 8.7), who were 70.9% females, 60.8% identifying as Black/African American (BA), and 36.8% residing in rural areas. The majority of clients had a prior HTN diagnosis (71.1%) and/or currently measured HTN (78.7%). Forty-three percent of adults screened had measured, diagnosed, and pharmaceutically treated HTN, while 31% had measured but untreated HTN. Black clients had higher measured systolic and diastolic BP and were more likely to also have been diagnosed with HTN (*p* < 0.05 for all). Linear regression indicated that lower systolic BP was predicted by not living alone (*p* = 0.003), White race (*p* = 0.004), and previous HTN diagnosis (*p* = 0.012), while female gender (*p* = 0.079) and decreasing body mass index (*p* = 0.053) had marginal predictive value. **Conclusions**: These results indicate that awareness and screening of HTN in this population are noteworthy, though management of the disease through ongoing screening and referrals is essential to reduce disparities.

## 1. Introduction

Hypertension (HTN) remains a critical public health concern, particularly among older adults in the southeastern United States, where prevalence rates are among the highest nationwide [1]. As a leading risk factor for cardiovascular disease, stroke, and mortality, HTN disproportionately affects minorities and socioeconomically disadvantaged populations, exacerbating health disparities in disease burden and outcomes [2,3,4]. Prior research has established that Black individuals and those residing in rural areas are more likely to experience both higher rates of HTN and poorer blood pressure (BP) control compared to their White and urban counterparts [5,6,7]. HTN prevalence is also strongly associated with lower socioeconomic status, with individuals of lower income facing higher risks of both HTN and related cardiovascular disease [8,9].

Furthermore, trends in HTN-related cardiovascular mortality indicate worsening disparities, particularly in communities with limited healthcare access and preventive screening programs [3,8]. Studies have shown that while BP control initially improved between 1999 and 2008, it has declined in recent years, disproportionately affecting Black individuals [7]. Additionally, untreated and uncontrolled HTN is linked to an increased risk of all-cause and cardiovascular mortality [4,10].

Given the persistent burden of HTN and the critical role of early detection and management in mitigating adverse health outcomes, community-based health screenings serve as an essential strategy for identifying individuals at risk and improving HTN awareness, diagnosis, and treatment [11]. The present study aims to assess the relationships among measured, diagnosed, and treated HTN in community-dwelling older adults in eastern Alabama who participated in student-led health screenings between 2017 and 2019. By evaluating BP measurements, medical history, and current prescriptions, we sought to assess HTN prevalence and BP control, with particular attention to differences across race, gender, and residential setting [12]. Understanding these patterns is crucial for guiding targeted interventions and shaping policy efforts aimed at reducing HTN-related health disparities in vulnerable populations.

## 2. Materials and Methods

### 2.1. Study Design

This observational study was conducted between 2017 and 2019 at 23 geographically distributed sites across eastern Alabama. Teams of medical and health profession students conducted comprehensive assessments to evaluate multidimensional health parameters among geriatric populations on 61 unique clinic days. Assessment domains encompassed physiological, pharmacological, nutritional, and psychosocial variables utilizing standardized measurement instruments.

Convenience sampling was employed through established partnerships with site administrators. Participant recruitment at congregate facilities was integrated within scheduled programming, while at residential facilities, recruitment occurred through strategic positioning of assessment stations in communal areas to encourage participation. Physiological parameter assessment (BP, blood glucose, and lipid profile) served as a primary recruitment facilitator. Inclusion criteria mandated English-language comprehension and provision of written informed consent. Exclusion criteria were not specifically delineated beyond consent capacity.

The interprofessional assessment teams comprised undergraduate and graduate students from multiple health science disciplines (social work, nursing, pharmacy, nutrition/dietetics, and medicine). Team members underwent standardized protocol training to ensure measurement reliability and received instruction in discipline-specific assessment techniques. Data collection procedures maintained participant anonymity post-consent. The investigation was determined to be not human subject research (NHSR) by the Auburn University Institutional Review Board (STUDY00000604, NHSR-5 May 2025).

### 2.2. Objective and Subjective Measures

The primary assessment instrument consisted of a multidimensional health screening tool [13]. A conversational approach allowed the groups to assess clients’ health literacy levels and obtain accurate information and data. The instrument incorporated dichotomous variables (yes/no), categorical variables, and continuous variables (age, time since medical screenings/procedures/vaccinations, etc.).

BP was measured using the auscultatory method with a calibrated sphygmomanometer. Clients sat quietly for 5 min with supported back and flat feet. The appropriately sized cuff was placed 2–3 cm above the antecubital fossa with the arm at heart level.

After palpating the brachial artery, the examiner positioned the stethoscope and inflated the cuff 30 mmHg above radial pulse disappearance. Pressure was released at 2–3 mmHg/second. Systolic pressure was recorded at the first Korotkoff sound (phase I) and diastolic at sound disappearance (phase V). Two measurements were averaged and recorded to the nearest 2 mmHg. All examiners were trained in this standardized protocol.

Pharmacological data collection included comprehensive medication reconciliation: when possible, dosage, frequency, duration, and therapeutic indications were obtained. In instances of incomplete medication knowledge, qualitative descriptions and therapeutic classifications were documented to facilitate post hoc identification. Medical history assessment followed systematic organ system methodology with terminology adaptations to maximize comprehension and data validity.

Anthropometric measurements included height (cm) and weight (kg), obtained via direct measurement using calibrated instruments when feasible or self-reported data when mobility limitations, facility constraints, or logistical impediments precluded direct measurement. Body mass index was calculated using the standard formula weight (kg)/height (m)^2^.

### 2.3. Covariates

Process variables included assessment site classification according to two taxonomic schemas: service delivery model and geographical location. Service delivery categorization comprised congregate facilities (senior centers, recreational venues, and food distribution centers [*n* = 7]) versus residential facilities (independent living communities, assisted living facilities, and age-restricted residential communities [*n* = 16]).

Geographical classification was defined by an urban–rural dichotomy, with urban designation (*n* = 15) assigned to locations within municipal boundaries of the three principal cities within the proximate Metropolitan Statistical Area as defined by the U.S. Census Bureau [14]. Spatial distribution analysis revealed a predominance of residential facilities in urban environments, while congregate facilities demonstrated greater prevalence in rural settings.

### 2.4. Statistical Analysis

Quantitative data analysis was performed using IBM SPSS Statistics version 28.0 (IBM Corporation, Armonk, NY, USA). Our hypothesis was that nationally observed disparities in HTN diagnosis and treatment would be observed in our sample of older adults.

Demographic and clinical characteristic differences were analyzed using Pearson’s chi-square test for categorical variables and independent sample *t*-tests for continuous variables. Systolic and diastolic BP differences among race and sex cohorts was examined via one-way analysis of variance (ANOVA) with Bonferroni post hoc corrections for multiple comparisons.

Predictors of systolic BP as a continuous variable were investigated using backward stepwise linear regression modeling. Statistical significance was established at α = 0.05 for all analyses.

## 3. Results

### 3.1. Participant Characteristics

The final analytical sample consisted of 357 adults aged 60 to 99 years with a mean age of 74.6 years (standard deviation [SD] = 8.7). The sample was predominantly female (70.9%), and the majority identified as Black (60.8%). Approximately one-third (36.8%) of clients resided in rural areas. The mean BMI of the sample was 29.1 kg/m^2^ (SD = 6.7), which falls within the overweight category as defined by the Centers for Disease Control and Prevention [15].

Regarding marital status, 41.4% of clients were widowed, 22.7% were single, 16.1% were married, 15.3% were divorced, and 4.5% were separated. Nearly two-thirds of clients (65.7%) reported living alone. Almost all clients (97.7%) reported having health insurance. The majority (69.4%) were assessed at congregate sites, while 30.6% were evaluated at residential facilities. Black clients were more likely to be female (75.6% vs. 63.6%, *p* = 0.017), more likely to be assessed at congregate sites (83.8% vs. 48.9%, *p* < 0.001), and more likely to reside in rural areas (47.0% vs. 22.3%, *p* < 0.001).

As shown in Table 1, 78.7% of clients had measured blood pressure values meeting hypertension criteria, defined as systolic BP ≥ 130 mmHg and/or diastolic BP ≥ 80 mmHg at the time of screening [16]. The mean systolic BP was 137.4 mmHg (SD = 17.3), and the mean diastolic BP was 80.1 mmHg (SD = 11.6), with the elevated hypertension prevalence primarily driven by increased systolic values exceeding clinical thresholds. A large proportion (71.1%) reported a prior diagnosis of HTN, and 58.8% were currently prescribed antihypertensive medications.

Black clients exhibited significantly higher mean systolic BP (140.1 mmHg vs. 133.1 mmHg, *p* < 0.001) and diastolic BP (81.1 mmHg vs. 78.6 mmHg, *p* = 0.042) than White clients. The prevalence of elevated BP (further reported as a categorical “measured HTN”) was also significantly higher among Black clients (83.4% vs. 71.4%, *p* = 0.008).

Notably, while Black clients were more likely to have a prior diagnosis of HTN compared to White clients (80.2% vs. 57.1%, *p* < 0.001), there was no significant difference in the proportion currently prescribed antihypertensive medications (59.9% vs. 57.1%, *p* = 0.660). This suggests a potential gap in treatment despite awareness of diagnosis among Black clients.

### 3.2. Race and Gender Disparities

**Figure 1:**
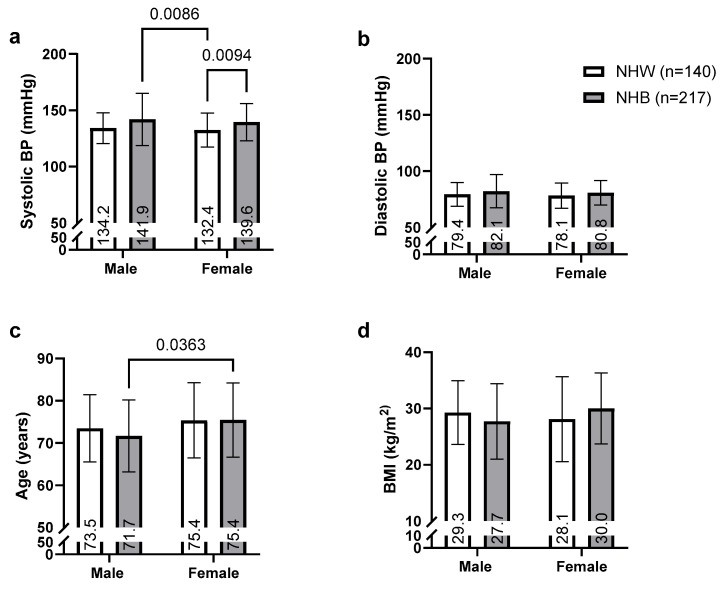
Race and gender comparisons of (**a**) systolic BP, (**b**) diastolic BP, (**c**) age, and (**d**) BMI. Means reported within bars. *p*-Values for multiple comparisons adjusted using Bonferroni correction.

Figure 1 presents a comparative analysis of systolic BP, diastolic BP, age, and BMI across race and gender categories. Figure 1a demonstrates significant racial disparities in systolic BP, with Non-Hispanic Black (NHB) clients exhibiting consistently higher values compared to Non-Hispanic White (NHW) clients across both genders. Among males, NHB clients had a mean systolic BP of 141.9 mmHg compared to 134.2 mmHg in NHW males (*p* = 0.0086). Similarly, NHB females exhibited higher systolic BP (139.6 mmHg) compared to NHW females (132.4 mmHg; *p* = 0.0094). This racial disparity pattern was consistent regardless of gender, with both NHB males and females exceeding clinical thresholds for HTN. In contrast, diastolic BP measurements did not differ significantly between racial groups within the same gender category (Figure 1b).

Figure 1c indicates age differences between groups, with a statistically significant difference observed between NHW males and NHW females (*p* = 0.0363). NHW females were notably older (mean age 75.4 years) than NHW males (mean age 73.5 years). No significant age differences were observed between racial groups within the same gender category, with NHB males (71.7 years) and NHB females (75.4 years) showing comparable age distributions to their NHW counterparts. No differences were observed in BMI between groups (Figure 1d).

### 3.3. Between-Race Comparisons of Systolic and Diastolic BP Measures by HTN Diagnosis and Treatment Status

**Figure 2:**
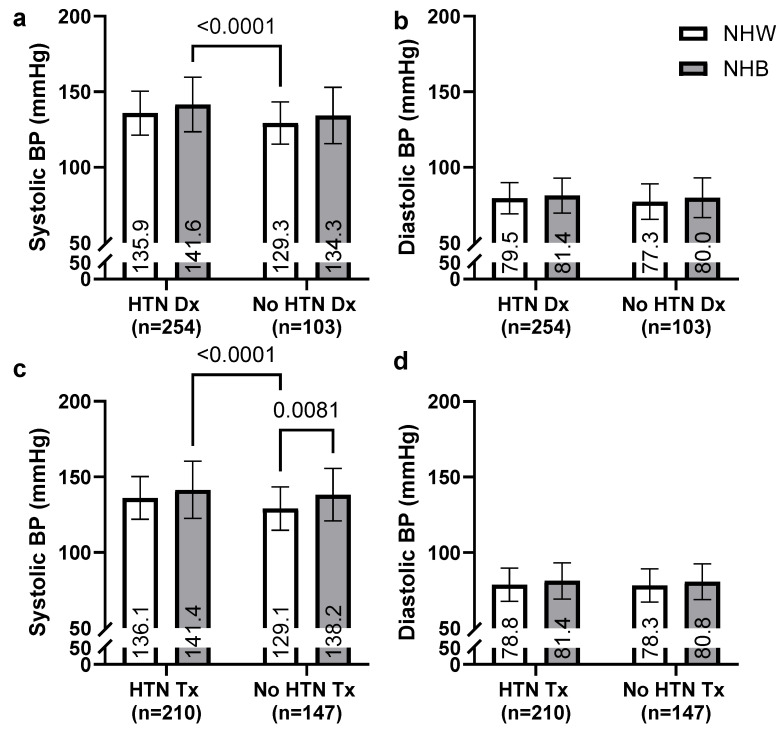
Race and gender comparison of systolic and diastolic BP by diagnosis (**a**,**b**) and pharmaceutical treatment (**c**,**d**) status. Means reported within bars. *p*-Values for multiple comparisons adjusted using Bonferroni correction.

Figure 2 illustrates BP disparities between races stratified by HTN diagnosis and treatment status. In Figure 2a, systolic BP was significantly higher in NHB clients with HTN diagnosis (HTN Dx) compared to NHW counterparts (141.6 mmHg vs. 135.9 mmHg, *p* < 0.0001). Diastolic BP measurements (Figure 2b) showed minimal differences between racial groups, with values ranging from 77.5 to 81.4 mmHg across all categories, without reaching statistical significance. The HTN diagnosis cohort included 254 clients, while 103 clients had no diagnosis.

When stratified by treatment status (Figure 2c,d), systolic BP remained significantly elevated in NHB clients receiving HTN treatment (HTN Tx) compared to NHW (141.4 mmHg vs. 136.1 mmHg, *p* < 0.0001). Among untreated individuals (No HTN Tx), NHB clients also exhibited significantly higher systolic BP than NHW (138.2 mmHg vs. 129.1 mmHg, *p* = 0.0081). Diastolic BP values showed consistent patterns across treatment groups (Figure 2d), with no statistically significant differences between NHB and NHW clients.

### 3.4. HTN Treatment Status and BP Control

**Figure 3:**
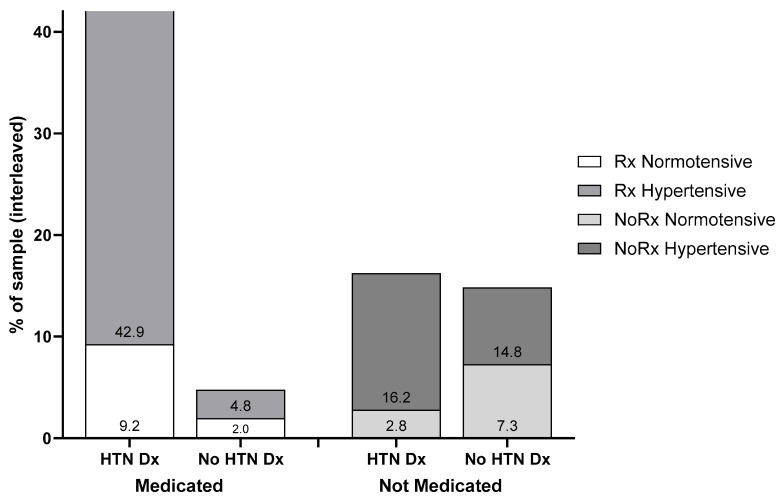
Distribution of clients across HTN diagnosis, treatment, and measured high BP categories at screening.

Figure 3 depicts the distribution of clients across HTN diagnosis status, medication status, and measured BP control. The largest proportion of the sample (42.9%) comprised individuals with diagnosed HTN who were receiving pharmacological treatment yet continued to exhibit elevated BP values during screening. This substantial group of clients with treated but uncontrolled HTN highlights a critical gap in disease management. In contrast, only 9.2% of the sample had both diagnosed HTN and pharmacotherapy with normalized BP readings, representing successful HTN control.

The 6.8% of clients reporting hypertensive medications with no diagnosis could have resulted from assuming controlled HTN equated to no disease or assuming a different purpose for the prescribed drug(s). Roughly four out of ten clients were not on hypertensive medications, though the largest share (31% of all individuals) were hypertensive and untreated. More concerning was that almost one in five (19% of all individuals) had an HTN diagnosis but were not currently taking medications for the disease.

### 3.5. Predictors of Systolic BP

Table 2 presents the results of backward stepwise linear regression analyses predicting systolic BP. The initial model included age, BMI, race, gender, HTN diagnosis, prescribed antihypertensive medications, marital status, living (alone) status, site type, and rural/urban location. After elimination of non-significant predictors, the final model (Model 6) identified five key predictors.

Lower systolic BP was associated with not living alone (β = −0.17, *p* = 0.003) and having a prior HTN diagnosis (β = −0.14, *p* = 0.012), suggesting potential benefits of awareness and treatment. Black race was associated with higher systolic BP (β = 0.16, *p* = 0.004). Female gender (β = −0.10, *p* = 0.079) and decreasing BMI (β = 0.11, *p* = 0.053) were marginally associated with lower systolic BP, though these relationships did not reach statistical significance at the *p* < 0.05 threshold.

## 4. Discussion

Herein we have described measured, diagnosed, and treated HTN in an ambulatory geriatric population in the southeastern US. The findings of this study highlight the persistent burden of HTN among older adults in this region, with notable racial and socioeconomic disparities in BP control. Despite a high prevalence of diagnosed HTN in our sample (71.1%), a substantial proportion of individuals exhibited uncontrolled BP, even among those receiving pharmacological treatment. These results align with national trends indicating stagnant or worsening BP control rates, particularly among Black adults and those residing in rural areas [17,18].

Racial disparities in HTN prevalence and control observed in our study are consistent with prior research demonstrating that Black individuals not only experience higher rates of HTN but also face greater challenges in achieving BP control, even with medical treatment [19,20]. This disparity has been attributed to a combination of biological, socioeconomic, and healthcare access factors. Lower-income individuals and those with limited healthcare access have disproportionately higher risks of uncontrolled HTN, as financial constraints and systemic barriers often limit their ability to adhere to prescribed treatment regimens [21]. Additionally, historical and ongoing structural inequities contribute to increased exposure to HTN risk factors such as chronic stress, inadequate healthcare access, and lower health literacy [22].

Our findings underscore the critical role of social determinants, particularly living arrangements, in influencing HTN control. Individuals living alone exhibited higher systolic BP in our sample, consistent with prior research linking social isolation to poorer HTN management [23,24]. Hu et al. found that socially isolated older adults were more likely to experience elevated BP and other cardiovascular risk factors, likely due to increased chronic stress, reduced physical activity, and less health-promoting behavior [25]. Golaszewski et al. further reported that loneliness and weak social ties were significantly associated with hypertension prevalence and poor cardiovascular outcomes among older women in the US [26]. Given that a significant portion of our sample reported living alone, and that the majority of participants were Black females, these findings suggest that limited social support may contribute to the observed disparities in HTN control.

Social support plays a crucial role in promoting medication adherence and health behaviors, which may explain the associations between living alone and elevated BP in our study cohort. Shahin et al. (2021) found that patients with hypertension who received consistent social support were significantly more likely to adhere to their medication regimens, suggesting that support systems can help mitigate forgetfulness, improve motivation, and reduce treatment-related stress [27]. Conversely, Hall and Heath noted that distrust in healthcare providers among Black patients was a major barrier to adherence, driven by historical injustices and personal experiences of discrimination [28]. Pugh et al. added that experiences of racism and lower trust in medical institutions correlated with poorer medication adherence in primary care settings [29]. Beyond individual health behaviors, social isolation limits both emotional well-being and practical access to healthcare, contributing to higher rates of uncontrolled HTN [25]. Addressing these challenges requires implementing structured social support interventions, such as community-based programs, peer mentoring, and provider-patient trust-building initiatives. These approaches may enhance HTN management outcomes, particularly for at-risk individuals facing barriers to healthcare access and adherence.

Moreover, urban–rural differences in BP control were evident in our sample, with rural participants demonstrating poorer HTN management. This is in line with national data showing that rural residents often experience lower HTN control rates due to reduced access to healthcare services, lower socioeconomic status, and health system limitations [18]. The American Heart Association recommends expanding access to self-monitoring tools like home BP monitors and delivering team-based care in trusted community settings—including barbershops, faith-based organizations, pharmacies, and home outreach visits—as a strategy to overcome geographic barriers and reach populations with limited access to traditional healthcare systems [22].

The high prevalence of treated but uncontrolled HTN in our sample underscores the need for enhanced clinical and public health strategies to improve BP control. Notably, 97.7% of clients in this study reported having health insurance, yet a substantial proportion still exhibited uncontrolled BP. This suggests that while healthcare coverage is essential, it alone may not be sufficient to ensure effective HTN management. Abdalla et al. (2023) emphasized the need for comprehensive, multi-component strategies that include team-based care coordination, where pharmacists, nurses, and community health workers engage directly with patients to manage medications and monitor BP [22]. Their research further highlighted the importance of structured care protocols, data sharing for treatment intensification, and integration of culturally tailored education and lifestyle interventions—including DASH diet promotion, physical activity, and stress management—as essential components of effective HTN control [22]. These interventions, especially when delivered in trusted community settings, should be widely adopted to address the multifaceted nature of HTN and improve BP control among underserved populations.

Finally, the clinical implications of uncontrolled HTN cannot be overstated. Uncontrolled BP is a well-documented risk factor for cardiovascular morbidity and mortality, with previous studies demonstrating a direct association between elevated BP and increased risk of cardiovascular disease and all-cause mortality [10]. Given the substantial proportion of individuals with uncontrolled HTN in our study, there is an urgent need for multi-faceted interventions that integrate pharmacologic and non-pharmacologic approaches to optimize BP control and mitigate the long-term health consequences of HTN.

### 4.1. Strengths and Limitations

This study also presents several important strengths. First, it was conducted in real-world community settings using student-led interprofessional teams, which provided both valuable outreach and rich observational data. The diverse and high-risk sample—predominantly Black, female, older adults, many of whom lived alone—offered meaningful insight into populations disproportionately affected by HTN. Second, the study collected comprehensive data across measured BP, diagnosis, treatment status, and social variables, enabling a multifactorial analysis of HTN disparities. Third, the integration of interdisciplinary student teams demonstrates a replicable model for future community health initiatives.

Despite these strengths, several limitations should be acknowledged. The cross-sectional design limits our ability to draw causal inferences between HTN status, demographic variables, and outcomes. The study also relied on self-reported diagnosis and medication use, which may introduce recall bias. Additionally, because the sample consisted of community-dwelling older adults participating in student-led screenings, findings may not generalize to broader or less-engaged populations. Lastly, while we assessed key social determinants of health, factors such as diet, physical activity, and psychosocial stressors were not measured and warrant further investigation.

### 4.2. Future Directions

Future research should focus on identifying and addressing the underlying factors contributing to persistent disparities in hypertension control, particularly among Black individuals, those living alone, and rural residents. A key area of exploration should be the integration of community-based participatory research approaches to develop culturally competent and patient-centered HTN management programs. Additionally, interventions should be designed to strengthen provider–patient relationships and improve trust in healthcare systems, which has been shown to influence medication adherence and treatment outcomes.

Building on the findings of this study, future research should explore tailored interventions aimed at improving HTN control in high-risk populations. Studies investigating the role of digital health interventions, such as telemedicine and remote BP monitoring, may provide insights into scalable strategies for improving BP control, particularly in rural and underserved areas also receiving technological support. Future research should examine the long-term effects of social support networks on HTN outcomes and assess interventions that enhance medication adherence and lifestyle modifications. Lastly, policy-level interventions addressing structural barriers to healthcare access, including affordability and healthcare provider availability, should be explored to mitigate disparities in HTN outcomes.

### 4.3. Conclusions

This study underscores the significant burden of hypertension among older adults in the southeastern United States, particularly among Black individuals, those living alone, and rural residents. The findings highlight ongoing disparities in BP control and the limitations of current treatment strategies in achieving optimal outcomes. Addressing these disparities requires a multi-level approach, including targeted community-based interventions, improved healthcare access, and enhanced support systems to facilitate long-term HTN management. By prioritizing equitable healthcare solutions and preventive measures, we can work toward reducing the public health burden of hypertension and its associated complications.

## Figures and Tables

**Table 1:** Participant characteristics.

		Total	Black	White	*p*-Value
		N (%)	N (%)	N (%)	
Gender	Female	253 (70.9)	164 (75.6)	89 (63.6)	**0.017**
	Male	104 (29.1)	53 (24.4)	51 (36.4)	
Age	Mean (s.d.)	74.6 (8.7)	74.5 (8.9)	74.7 (8.6)	0.870
BMI	Mean (s.d.)	29.1 (6.7)	29.5 (6.5)	28.5 (6.9)	0.218
Systolic BP	Mean (s.d.)	137.4 (17.3)	140.1 (18.3)	133.1 (14.6)	**<0.001**
Diastolic BP	Mean (s.d.)	80.1 (11.6)	81.1 (11.9)	78.6 (11.0)	**0.042**
Measured HTN	Yes; N (%)	281 (78.7)	181 (83.4)	100 (71.4)	**0.008**
Diagnosed HTN	Yes; N (%)	254 (71.1)	174 (80.2)	80 (57.1)	**<0.001**
Prescribed HTN meds	Yes; N (%)	210 (58.8)	130 (59.9)	80 (57.1)	0.660
Marital status	Single	80 (22.7)	47 (21.9)	33 (23.9)	0.097
	Married	57 (16.1)	29 (13.5)	28 (20.3)	
	Separated	16 (4.5)	14 (6.5)	2 (1.4)	
	Divorced	54 (15.3)	32 (14.9)	22 (15.9)	
	Widowed	146 (41.4)	93 (43.3)	53 (38.4)	
Living status	Alone	230 (65.7)	133 (62.7)	97 (70.3)	0.167
	Not alone	120 (34.3)	79 (37.3)	41 (29.7)	
Insurance	Insured	341 (97.7)	210 (98.1)	131 (97.0)	0.716
	Not insured	8 (2.3)	4 (1.9)	4 (3.0)	
Screening site	Congregate	234 (69.4)	166 (83.8)	68 (48.9)	**<0.001**
	Residential	103 (30.6)	32 (16.2)	71 (51.1)	
Location of site	Rural	124 (36.8)	93 (47.0)	31 (22.3)	**<0.001**
	Urban	213 (63.2)	105 (53.0)	108 (77.7)	

Bold *p*-values are statistically significant via independent samples *t*-tests for continuous variables and chi-square tests for categorical variables.

**Table 2:** First and last backward stepwise regression models predicting measured systolic BP in 357 community-dwelling adults *.

Model	Factor	Unstandardized. Coeff.				
		B	Std. Error	Beta	t	*p*-Value
1	(Constant)	135.77	13.63		9.96	**<0.001**
	Age	0.08	0.11	0.04	0.704	0.482
	BMI	0.31	0.15	0.12	2.08	**0.038**
	Race	5.24	2.13	0.15	2.464	**0.014**
	Gender	−4.06	2.14	−0.11	−1.901	0.058
	HTN diagnosis	−4.34	2.45	−0.11	−1.774	0.077
	HTN meds Rxed	1.87	2.21	0.05	0.848	0.397
	Married	−0.91	3.07	−0.02	−0.296	0.768
	Not living alone	−5.99	2.52	−0.17	−2.372	**0.018**
	Site type	−2.38	2.61	−0.06	−0.912	0.362
	Rural or urban	1.28	2.23	0.04	0.574	0.567
6	(Constant)	141.71	7.82		18.123	**<0.001**
	Not living alone	−6.01	1.99	−0.17	−3.013	**0.003**
	Race	5.65	1.94	0.16	2.908	**0.004**
	HTN diagnosis	−5.40	2.15	−0.14	−2.513	**0.012**
	Gender	−3.69	2.09	−0.10	−1.763	0.079
	BMI	0.28	0.14	0.11	1.946	0.053

* For binary variables, the following are listed in order of increasing value in the regressions: Race—White, Black; gender—male, female; HTD diagnosis—no, yes, HTN meds Rxed—no, yes; married—no, yes; not living alone—alone, not alone; site type—congregate, residential; rural or urban—rural, urban.

## Data Availability

Data is available from the corresponding author upon reasonable request.
